# The Best Way to Create a Guideline

**DOI:** 10.21470/1678-9741-2021-0954

**Published:** 2021

**Authors:** Eduardo Augusto Victor Rocha, Henrique Murad

**Affiliations:** 1 President of the Brazilian Journal of Cardiovascular Surgery, São Paulo, São Paulo, Brazil.; 2 Universidade Federal do Rio de Janeiro, Hospital Universitário Clementino Fraga Filho, Rio de Janeiro, Rio de Janeiro, Brazil.

The need to practice Evidence-Based Medicine encourages us to search for the best clinical practices. The treatment guidelines have become a synthesis of scientific evidence for the use of the common physician. To properly organize these guidelines, medical societies seek the best specialists who preferably base their opinions on multicenter and randomized studies. When there are no substantiated articles for a specific issue, they use expert´s opinion with extensive experience and knowledge to establish the best practices. For these guidelines to be respected, a plural discussion with different opinions is necessary. A collection of antagonistic opinions with scientific basis leads to increasing knowledge and the opening of new fields of research. Health care payers (private or public) have started using these guidelines to substantiate indications and contraindications for medical procedures, with enormous influence on the daily lives of cardiologists, interventionalists, and cardiovascular surgeons.

In October 2020, the Brazilian Society of Cardiology (BSC) published in the Brazilian Archives of Cardiology the Update of the Brazilian Guidelines for Valvular Heart Disease - 2020^[1]^. It was prepared by professionals of recognized qualification and experience, however the participation of the Brazilian Society of Cardiovascular Surgery (BSCVS) was not requested. The BSCVS is an independent society, being one of the 54 societies recognized by the Brazilian Medical Association, and its partners are responsible, since the 1950's, for the surgical treatment of patients with valvular heart disease.

Large cardiology and cardiovascular surgery societies, such as the American and European societies, work together to create their guidelines^[2,3]^. Thus, we are reflecting on the inadequacy of the way in which this guideline was created and published. In this same issue of the Brazilian Journal of Cardiovascular Surgery, questionable studies of the three most recent guidelines on valvular heart diseases are shown^[4]^.

We would like this editorial to show our concern with the non-invitation of BSCVS to assist in the development of this Brazilian guideline on valvular heart disease and also to present the way that we consider appropriate for the scientific development of both societies, always aiming at the best assistance for our patients.
